# An examination of the Petroleum Industry Act 2021: prospects, challenges, and the way forward

**DOI:** 10.12688/f1000research.132539.1

**Published:** 2023-05-25

**Authors:** David Oladeji Ehijie Borha, Olusola Joshua Olujobi

**Affiliations:** 1 Department of Public and International Law, College of Law, Afe Babalola University, Ado Ekiti, Ekiti State, Nigeria

**Keywords:** Petroleum Industry, Oil and gas, Petroleum Industry Act, Prospects, Challenges

## Abstract

**Background:** The study examines the gaps in the provisions of the Petroleum Industry Act (PIA) that could hinder the effective application of the Act in attaining its objectives. The repealed Petroleum Act of 1969 became obsolete and largely incapable of meeting the emerging global best practices in the industry due to inadequate sanctions, failure to address the aspirations of the people of the oil-bearing states, among others. Hence, the need for the PIA of 2021 to overhaul the industry to meet global standards though some controversial provisions that pose challenges to its proper implementation.

**Methods:** The study examines the PIA to identify its prospects, challenges, and the way forward. The methodology the study utilises is doctrinal research with reports from existing literature and tertiary data sources such as newspapers, the Internet, and websites. Pertinent data collected from these sources were theoretically analysed and argued with current literature on the subject.

**Results:** The finding is that the PIA does not make adequate provisions for the energy transition in line with Nigeria’s Nationally Determined Contributions (NDC) under the Paris agreement; the PIA was provided for weak institutions, which translates to weak implementation and enforcement of the law which further widening the gap between the law and reality.

**Conclusions**: The study concluded that, although the Act delivered the much-needed stability in the petroleum industry in Nigeria, there is a need for an overhaul of the Act to further protect the interest of host communities and allow for co-ownership of petroleum resources by the state government.

## Introduction

Nigeria is blessed with numerous extractive resources such as crude oil, natural gas, gold, limestone, granite, and coal, among others. Hydrocarbon resources are some of the most valuable due to their high demand globally. Nigeria is currently the second-largest crude oil producer and one of the world’s top gas producers, possessing one of the Africa’s biggest gas reserves. ^1^ Nigeria is set to benefit from the global demands for gas as a result of the ongoing conflict between Russia and Ukraine, ^2^ as it is expected to benefit from the gaps in the global demands for gas and other supply chain caused by the ongoing war, hence the need to further overhaul the law.

The study examines the Petroleum Industry Act (PIA) to identify its prospects, challenges, and the way forward. The repealed Petroleum Act of 1969 appears obsolete and largely incapable of meeting the emerging global best practices in the industry due to inadequate sanctions, failure to address the aspirations of the people of the oil-bearing states due to a lack of measures that would have directly addressed the socioeconomic and environmental challenges affecting them, due to the detrimental effects of oil and gas exploration activities among others. ^3^

The economy of Nigeria is heavily dependent on its hydrocarbon resources; however, as a result of the global transition to low-carbon energy sources, there is a need for Nigeria to look inward. ^4^

On August 16, 2021, President Muhammadu Buhari signed the Petroleum Industry Bill (now the act) into law as the principal legislation regulating Nigeria’s oil and gas industry. Without a doubt, the PIA of 2021 is a landmark improvement in Nigeria’s oil and gas industry. Nonetheless, the act still has some flaws such as the recurring conundrums of gas flaring that have not been adequately addressed by the act. ^5^ Likewise, the need for an energy transition in the context of the global demand and campaign against fossil fuels in an era when decarbonization is a global necessity.

The need for reform of the legislative and regulatory challenges confronting the act and to take full advantages of its prospects necessitate this study to bring the Nigeria’s petroleum legal in conformity with best practices and international treaties and agreements. The study will answer the following research questions:

a. What are the remedies to the gaps identified in the provisions of the PIA that could hinder the effective application of the act in attaining its objectives?
b. What potential does the new PIA 2021 have in tackling the challenges in Nigeria’s petroleum industry when compared with international best practices?
c. What are the key policies in Qatar, Kuwait and Norway’s oil and gas framework, that may be replicated in the Nigerian oil and gas regime?

The study will be useful to the Federal Government of Nigeria, policymakers in the oil and gas sector, energy practitioners, researchers, and scholars in policy formulation and enactment of legal regime regulating the oil and gas industry by offering guidelines for legislators on the best approach to employ when drafting a sustainable oil and gas legal framework to promote transparency, energy diversification by incorporating renewable energy and other low-carbon energy sources into the nation’s energy mix, and active citizen participation in the development of low-carbon energy technologies being a global clarion call for low-carbon energy utilisation.

The study is organized into five sections, with the introduction and statement of the problem in the first section. Section two is the literature review, including resource ownership theory and utilitarian theory. Section three outlines the methodology. Section four presents the results by deliberating on the challenges of the PIA 2021, analysing of the oil and gas legal regime in Qatar, Kuwait and Norway, and presenting other findings of the study. Section five contains the discussion, conclusions and recommendations.

### Statement of the problem

Preceding the discovery and production of crude oil in Nigeria, no detailed law had been enacted for the fiscal, regulatory, and administrative regime in the petroleum sector. Subsequent enactment of laws aimed at governing the petroleum sector was seen to be inefficient. The legal framework before the PIA lacked transparency and accountability due to its archaic nature, thus it was found ineffective and not in conformity with current international best practices and did not reflect the current economic reality. To address some of the flaws in the repealed Petroleum Act 1969 and the challenges in the Nigerian petroleum industry, the PIA 2021 was enacted. ^6^ The PIA, though viewed as a panacea to the challenges in the petroleum sector, continues to be riddled with corruption, poor governance and poor management reflected by the appalling state of refineries capacity in Nigeria. ^7^ This in turn occasions the importation of petroleum products with subsidy payment which gives room for endemic corruption in the sector. Crude oil theft issues and lack of effective metering have also been seen to be some of the challenges affecting petroleum production and national revenues, this is also exacerbated due to lax regulatory enforcement by relevant agencies. Nigeria has incurred a loss of over $ 2.1 billion as a result of oil theft. ^8^

## Literature review

According to Umenweke and Chukwuma, ^9^ the mandate of the PIA is to assist the government in attaining 40 billion barrels of reserves and production levels of four million barrels per day. This mandate was merely stated; they failed to state precisely how the act could assist the government in attaining this goal. Furthermore, the authors merely stated that the PIA now curtails the powers of the Minister in approving or revoking an oil license, without precisely stating the nature of the curtailment and the applicable sections of the act. This is one of the gaps in the existing literature that this study intends to fill among others, this can be done by stringent enforcement or implementation of the act’s provisions by its regulatory authorities with the backing of the executive arm of government and the strong political will of the president.

Umenweke and Chukwuma noted that there is nothing in the act to show adequate commitment to climate change and energy transition. This position may also not be accurate because there are provisions in the act on the utilisation of the natural gas potential of the country. For instance, Section 52(1) of the PIA establishes a Midstream and Downstream Gas Infrastructure Fund (‘the fund’), which shall be a body corporate with perpetual succession and a common seal and reside in the authority as prescribed by this act. It shall have a governing council which shall supervise and make investment decisions for it. It shall be financed primarily from the 1% levy on the wholesale price of petroleum and natural gas sold and produced. It is expected that the fund shall be used to make equity investments in infrastructure related to midstream and downstream gas operations to increase the domestic consumption of natural gas in Nigeria in projects which are financed in part by private investment; encourage private investment; reduce or eliminate gas flaring. However, the provisions of the PIA are not adequate to tackle the issue of climate change given that even the role of gas in the energy transition mix and its production still have adverse effects on global warming and climate change.

### Theoretical framework

The domanial ownership theory states that the sovereign possesses ownership of petroleum and other natural resources. According to this view of sovereign ownership, the state would have complete control and permanent sovereignty over all-natural resources, including petroleum. ^10^ This ownership theory is considered to be the most prevailing theory on the ownership and control of petroleum resources. With the notable exception of the United States of America, this theory is favoured in Nigeria and virtually every other country with natural resources. The sovereign, state, or government owns and controls all mineral wealth in the country. These ownership and control rights are primarily established in the constitutions of most countries. Section 1 of the PIA confers the whole ownership and control of any petroleum in, under, or upon any land in Nigeria in the State. This authority allows the state to either explore resources directly or provide rights to third parties on any terms it considers appropriate. ^11^

### Resource curse theory

The endowment of natural resources has been described as a desirable benefit to the state and the people living within its geographical boundaries. The expected benefits of such endowment, however, have not always materialized, primarily because of several factors, such as the failure to use the revenues generated from the exploitation of natural resources to promote development, the abrogation of good governance, corruption, and a lack of accountability. This results in a “paradox of plenty”, with glaring underdevelopment and poverty in the presence of abundant resources. As a result, a state with abundant natural resources suffers from crippling underdevelopment, social unrest, and a lack of public accountability. ^12^ The resource curse is a phenomenon that is pervasive in Nigeria and continues to be the country’s biggest obstacle to socioeconomic development. This is also a subject that has gained a lot of attention in the literature.

### Theory of utility

Utilitarianism is a moral and ethical philosophy in political theory. ^13^ Although it has its roots in Greek philosophy, it gained popularity because of the work of Jeremy Bentham and J.S. Mill (1748-1832). One of the strongest and most convincing theories of normative ethics in the history of philosophy is utilitarianism. Though not fully articulated until the 19th century, proto-utilitarian positions can be discerned throughout the history of ethical theory. The principle of utility is described by Bentham as ‘the principle that approves or disapproves of every action, according to the tendency that it appears to have to increase or decrease the happiness of the party whose interest is in question. The challenges affecting the Niger Delta communities are brought to light by a review of how the Nigerian government’s policies concerning oil and gas management are influenced by utilitarian dictates, which affect the host communities, while the Niger Delta communities continue to experience slow development. ^14^

Another criticism levelled against the PIA is the meagre 3% allocation to the host communities, rather than the 10% that was demanded by the communities. This refers to the agitation of the Pan Niger Delta Forum (PANDEF) regarding the meagre 3% provision for the Host Communities Development Trust (HCDT) Fund and the allocation of 30% of Nigerian National Petroleum Corporation Limited (NNPC Ltd.) profit for the Frontier Oil Exploration Fund. The current researcher agrees with the authors that this 3% allocation to the host community has been condemned by activists, civil society organisations and host communities for failing to adequately cater for the host communities. ^15^

Similarly, Bielu ^16^ among other things, criticised the provision of a 3% contribution for the host community but 30% for the frontier Basin as being unfair. He also contended that due to the existence of the Niger Delta Development levy coupled with the 3% levy for the host communities there is a need for aggregation of the levies or scraping of one. This study also agrees with the author’s view that the PIA can benefit from an amendment to bring it to conformity with other legislations.

Blythe and Todd ^17^ also examined the fiscal changes introduced by the PIA. They rightly stated that the petroleum profits tax has been replaced by the hydrocarbon tax (HCT) and the companies’ income tax (CIT) and that the HCT only applies to crude oil, condensates and natural gas liquids produced from associated gas. However, they failed to state that this only applied to companies in the upstream oil and gas operations in onshore, shallow water and deep offshore. The authors stated the tax rates for HCT but failed to state the tax rate for CIT. This is also a gap in the existing literature that this current study intends to fill.

Mekwunye, ^18^ similarly, on the issue of transparency in the petroleum industry, posited that the lack of transparency and accountability in the Nigerian petroleum industry is so pervasive that Nigeria is unable to account for the amounts of crude oil produced, exported, or the receipts of sales proceeds.

## Methods

The doctrinal research methodology will be adopted in this research. In carrying out this library-based research, primary and secondary sources of law and relevant existing literature will be consulted and relied upon, using sources obtained from internet and physical library searches for ‘Petroleum Industry Act 2021’ or ‘PIA’. Reliance will be placed on an examination of primary legal sources, including case law, judicial precedents, and domestic and international legislation, including the Constitution of the Federal Republic of Nigeria 1999 (as amended 2011), the PIA 2021, the repealed Petroleum Act 1969, the repealed Petroleum Profits Tax Act as well as other relevant statutes. Lessons will also be drawn from international legislation, including Law No 10 1974 of the establishment of Qatar’s petroleum, Law No 6 1980 of the establishment of Kuwait’s petroleum and Norway’s Petroleum Act 1996, as well as other enactments. These countries laws are chosen as a model because they comply with the international best practices on the regulation of petroleum industry as a comparison to Nigeria’s petroleum laws.

## Results

### Prospects of the PIA 2021

The prospects of the PIA are, among others, the introduction of a new regulatory and governance structure, the creation of new types of licences, the commercialization of NNPC Ltd., and the introduction of a new fiscal framework. These will be comprehensively examined. Some of the challenges of the PIA such as the allocation of 30% to the frontier basins fund, allocation of just 3% to the host communities; use of ambiguous and complex words, and lack of whistle-blowing provisions will also be comprehensively analysed. ^19^ The act repealed approximately 10 existing petroleum laws governing the petroleum industry before the enactment of the act. The repealed Petroleum Act lacked measures that would have directly addressed the socioeconomic and environmental challenges affecting the inhabitants of the host communities as a result of the detrimental effects of oil and gas exploration by foreign and Nigerian oil corporations. The act establishes NNPC Ltd. as a profit-oriented legal entity. NNPC Ltd.’s new status as a limited liability company under the Company and Allied Matters Act (CAMA) 2020, the entity may be able to benefit from the capital market by being listed after satisfying the listing requirements and it raises capital for its operation by allotting its shares to the public. The act unbundled the industry by designing a well-organised governance structure with dynamic and distinct roles for the industry. The act further overhauls the fiscal and tax regime of the petroleum industry. It decreases the tax and royalty rates that operators must pay to encourage investments from both domestic and foreign investors.

### The challenges of the PIA 2021

The act introduces and specifies the regulations, procedures, and institutions which will ensure good governance, transparency, and accountability in the petroleum industry. However, there can never be perfect legislation. Certain provisions of the PIA have been deemed contentious, while others have been deemed potentially problematic that require legislative amendment to promote international best practices in the industry. ^20^

*Ownership of natural resources*

Section 1 of the PIA, which deals with the ownership of petroleum by the Federal Government of Nigeria, should have been drafted to include the state, Local Governments, and the host communities in addition to the Federal Government as owners of the petroleum in the country. The three tiers of government and the host communities need to have been included in the vesting right to reduce incessant agitation for resources control, pipeline vandalization, crude oil theft and other oil related crimes. This is to reduce the Federal Government’s exclusive rights and total sovereignty over petroleum resources as it is practised in the United States and Canada.

*North and south division*

In an effort to address the industry’s long-standing issues, the PIA 2021 created unintentional divisions or factions between the north and the south legislatures in the National Assembly. The bill was opposed by lawmakers and prominent leaders from the oil-rich Niger Delta states, and many southern legislators believe it favours the northern interests at the expense of the southern interests. According to the southern legislators, the law has been modified to benefit the northern part of the country. The southern lawmakers now believe that they are being cheated by the northern legislators. The host communities are also dissatisfied since they asked for 10% but the lawmakers rejected it. Pan-Niger Delta Forum (PANDEF), on the other hand, had also urged the lawmakers to immediately reverse the 3 percent they allocated to the host communities. ^21^

In reality, the PIA still incites hostility in the Niger Delta. The Host Community Development Trust Fund (HCDTF)’s percent contribution, according to the PIA’s critics, is insufficient, while NNPC Ltd.’s 30% profit for the Frontier Basin Development Fund is unfair. Public remarks by certain Northern politicians have lent credibility to the Southern suspicions that the Frontiers Basin Fund is a tool for redistributing resources to the North. For instance, a northerner who works as the Group Managing Director of NNPC Ltd. recently claimed that the law will be more advantageous to the north since new crude oil reserves are being discovered in the region and the revenue derivable from exploration would accelerate additional discoveries in the north. Such utterances undermine efforts to get a national consensus on the petroleum policies that are interpreted in a way that is impartial to regions and that is in the national interest. ^22^

*Double taxation*

Oil companies are required to pay a HCT under section 260 of the PIA. The same companies must also pay the CIT. Under section 302 of the act ^.^ These taxes may be seen as double taxation by the companies and investors may also be discouraged from investing in Nigeria’s petroleum industry by such taxation requirement.

*Ambiguous wording and imprecise language*

The difficulty interpretation and legal ambiguities associated with the law is one of the biggest challenges of the act. For instance, it is not apparent if the duties of the host community development trust are separate from or in addition to current community levies such as the Niger Delta development levy. Similar to this, the act is silent regarding the definitions of “frontier basin” and “host community,” referring to the NUPRC for a definition of the former and settlors or licence holders for the definition of the latter. Frontier Basin Exploration does not have a proper definition under the PIA. These definitions affect revenue; they are not revenue neutral. This ambiguity breeds controversy and even the possibility of litigation, especially where stakeholders define them differently. ^23^

*Tensions over oil revenue sharing*

The PIA has significant effects on the federation’s revenue as well as those of the states and the local governments. First, the three tiers of governments may see a significant drop in revenue accrued to them as a result of the reduction in taxes and royalties. Any revenue generating entity owned federation must deposit its profits in a fund called the Federation Account so that the three arms of governments can share them. ^24^ More than 80% of the funds received by the states and local governments come from the Federation Account. Therefore, NNPC Ltd.’s contribution to the Federation Account may be significantly reduced as a result of the requirement that 30% of its revenues be set aside for frontier exploration. The Federation Account’s shared revenues with the different tiers of governments will decline in the near future. Numerous states and local governments, particularly those with extremely limited internal revenue-generation capabilities, will not be able to fulfil their responsibilities to provide social services to their populace. On the other hand, such a move would spur innovations at the state and local government to boost internal revenue-generating capacity and fiscal efficiency, therefore, this policy may have a good long-term outcome on the (3) three ties of governments. ^25^

The success of the operations in Nigeria’s petroleum industry depends significantly on the host communities. ^26^ The partnership may be the key to achieving profitable returns from the most capital-intensive ventures. However, throughout time, there has not been a friendly relationship between host communities and petroleum corporations. ^27^ Petroleum operations have often been interrupted by the hostility and conflict. The significant theft of crude oil, the destruction of pipelines, and the ongoing suspension of large oil fields may also have been caused in part by the conflicts.

The government’s inability to satisfy its Organization of the Petroleum Exporting Countries (OPEC) crude oil output quota of around 1.8 million barrels per day in production has recently averaged 1.3 million to 1.4 million barrels per day, which indicates the severity of the issue. The tense relationship may in part be ascribed to these regions’ sluggish rate of growth. The government has attempted to solve this issue on several occasions with efforts that have varied in their degrees of effectiveness. Among these programmes is the establishment of the Derivation Fund, which is financed with 13% of oil revenues from the Federation Account and distributed to oil-producing regions; the establishment of the Niger Delta Development Commission (NDDC) in 2000, with an NDDC Levy of 3% of an overall annual budget of oil producing firms (Niger-Delta Development Commission [Establishment etc.] Act 2000 Act No 6 LFN). The establishment of the Ministry of Niger Delta Affairs in 2008, with a total budget allocation of NGN 584.6 billion between 2008 and 2022. and a number of others. In another effort to remedy the strained relationship between the host communities and petroleum corporations, the PIA mandated that petroleum industry operators to create a HCDT, as previously mentioned. The HCDT is a fund that will be established for the benefit of communities in the areas of operations of the petroleum corporation’s activities. The fund will support host communities’ infrastructure development and economic development and empowerment. The PIA allows operators to use their discretion when determining the host communities to include additional communities that may have an indirect effect on the HCDT’s success, although these other communities are not ’appurtenant’ to the operator’s area of operations ^.^ The PIA lays forth the formation of petroleum corporations’ development of the host communities activities with regard to the host communities, including the establishment of trusts for the domicile of companies’ contributions to community development. ^28^

The host communities, on the other hand, are still disgruntled with the PIA’s requirement that petroleum corporations allocate 3% of their annual operational expenditure in the immediately preceding calendar year to the HCDTF, when they had requested at least 10%. Moreover, because indigenous oil firms hold the majority of onshore oil wells, host communities are unsure if this contribution will be given at all. The prior owners of the oil wells were foreign corporations, which meant that there were two sets of laws to ensure legal compliance Nigerian and those of the home country. With internal ownership of the majority of onshore oil wells, the risk of non-compliance with the host community contributions is high, particularly in Nigeria, where the judiciary is seen as weak, and court judgments are rarely upheld and political control of the industry is pertinent, because indigenous corporations dominate the onshore oil industry, there are legitimate concerns that the HCDTF will likely not receive any funding, which would leave the host communities underdeveloped. ^29^

*Lacunas in the HCDT provisions*

A few provisions have been discovered to be omitted from the HCDT under the PIA, some of these include:

i. The midstream operators’ required contribution to the HCDT is not addressed by the PIA.
ii. A procedure for resolving disputes between the host communities and the operators does not exist under the PIA.
iii. There is no apparent framework for cooperation between the operators who may cover the same host community in order to avoid duplication of projects and achieve synergy.

### Corruption

It’s widely understood that corruption has plagued Nigeria ever since the country gained its independence in 1960, corruption persists now in every sector of the country’s economy, and unless it is addressed and eliminated at all levels. ^30^ Corruption is an ever-present problem in the petroleum industry, from the initial stages of exploration and drilling through the latter stages of refining and distribution. Where a country mainly dependent on one single product or resources, like petroleum, for its social, economic growth, and development and corruption in that industry has a cumulative effect on the rest of the country’s economy and other industries.

The PIA is expected to attract investment and to boost production, but these cannot adequately come into play if the stakeholders do not work impartially by prioritising the interest of the nation over political apathy. Provisions of the law are ultimately intended to improve transparency in the petroleum industry while ideally significantly reducing revenue losses due to corrupt or sharp practices and lack of accountability in the sector. The Government of Nigeria’s revenues from petroleum products has decreased due to corruption.

It is alleged that government officials are responsible for this corruption and the misuse of oil revenues. ^31^ Officials in the government’s petroleum industry have been accused of taking bribes from upstream petroleum corporations in exchange for illegal contract awards.

### Analyses of the oil and gas legal regime in Qatar, Kuwait, and Norway

*Qatar in perspective*

The cornerstone of the Qatari economy is petroleum. ^32^ The largest non-associated natural gas field and substantial oil reserves in the world are situated in Qatar. The country is a prominent stakeholder in the global energy market, the country is at the forefront of the gas-to-liquids and liquefied natural gas sectors, and quickly emerging as a key hub for hydrocarbon research and development. ^33^ Until recently, Qatar was a member of OPEC and has had a significant impact on global energy markets since it assumed the Organization of Arab Petroleum Exporting Countries’ rotating annual presidency in 2015. Even as efforts to diversify the economy are made, the industry continues to dominate the national economy. The petroleum resources of Qatar account for over 70 percent of the country’s total revenue; 60 percent of the country’s gross domestic product and more than 85 percent of its export revenue come from the petroleum industry. ^34^

Most of Qatar’s hydrocarbon needs are satisfied by domestic production, however, limited volumes of oil with specified requirements are imported for a few purposes. As of 2022, Qatar’s oil reserves were 25.2 billion barrels, while its oil production dropped to 1.9 million production, and transport agreements, Qatar Energy serves as the government’s commercial arm. Similar to Nigeria, NNPC Ltd. is an independent commercial legal entity that operate like other business entity with statutory obligations such as payment of taxes to the Federal Government and declaration and payment of dividends to its shareholders if any. The state owns all-natural resources, including oil and gas similar to the practice in Nigeria under Section 44(3) 1999 Constitution of the Federal Republic of Nigeria (as amended). production, and transport agreements, Qatar Energy serves as the government’s commercial arm.

Qatar has enacted numerous laws regulating its natural resources, these include the following among others:

i. Law No. 10 of 1974 (as amended by Law No. 15 of 1988) established Qatar Petroleum (now Qatar Energy), the national oil and gas company.
ii. Law No. 4 of 1977 on the conduct of petroleum operations and the conservation of petroleum resources in Qatar.
iii. Law No. 3 of 2007, deals with the exploitation of natural resources, which includes mining, oil and gas operations, and any related activities. (Exploitation of Natural Resources Law).
iv. Law No. 15 of 2007 awarding Tasweeq the sole right to sell and export petroleum produced in Qatar.
v. Law No. 11 of 2012 giving Muntajat the sole authority to promote and market chemicals and petrochemicals produced in Qatar.

In Qatar, Qatar Energy has the sole and absolute right to explore for, produce, invest in, and develop any hydrocarbons, including petroleum, natural gas, and all others. Any corporation may apply for a licence or authorization from Qatar Energy to conduct petroleum operations within the country. Prior to undertaking any petroleum operations, a contractor commitment to provide Qatar Energy with a comprehensive overview of the proposed operations. This description covers plans, location, production capacity, operating modes, engineering data, cost estimates, varied computations, and other relevant documents, information, and statistics. Exploration and Production Sharing Agreements (ESPAs) and Development and Production Sharing Agreements (DPSAs) often provide for the formation of a management committee comprised of equal participation from the government and the contractor. the management committee has the capacity to assess and approve budgets and work plans, it contributes to concession-level decision-making on proposals for the submission of development plans, ceding of non-producing areas under the requirements of the EPSA/DPSA, and delineation of development areas. Under an EPSA, the contractor must submit a development and production programme and budget on commercial discovery and annually afterwards, outlining the development and production operations which the contractor plans to carry out throughout the year and any projected expenditure. The management committee must approve and may revise the development and production programme and budget. ^35^

The Exploitation of Natural Resources Law requires Qatar Petroleum’s authorization before granting concession rights or transferring or assigning concession holders’ rights to third parties. Leases, licences, and concessions for the exploitation of hydrocarbons are granted following a decision by Qatar Petroleum and the State of Qatar. Previously, these agreements were made between Qatar Petroleum and one or more multinational petroleum corporations. using an instance of the Al Shaheen Field, when former Qatar Petroleum formed a joint venture with Total, and the government granted the concession to the new joint venture corporation. ^36^

Decommissioning requirements will be outlined in the applicable licence/concession agreement; however, these clauses typically result in additional agreement with Qatar Petroleum. The decommissioning must be consented to by the parties and Qatar Energy if the agreement does not address it. The Environmental Protection Law’s provisions will still apply to the parties, and any breach of them will subject them to penalties under the law. For this, a contractor often presents a plan for the abandonment, decommissioning, and disposal of any substantial fixed and mobile assets employed in conjunction with the conduct of the petroleum activities and including the restoration of site locations. The plan also specifies the duration for implementing each component as well as the expected decommissioning expenses. Sometimes a decommissioning committee will be created to oversee the technical and fiscal aspects of abandoning, decommissioning, and disposing of fixed and moveable assets. It will also be responsible for approving any decommissioning plans submitted by the contractor to the management committee. In most cases, the contractor is also in charge of removing, abandoning, decommissioning, plugging, and salvaging facilities and wells. In Nigeria, sections 232(1)-(14), 233(1)-(12) of the PIA 2021 and the provisions of the National Oil Spill Detection and Response Agency (Establishment) Act 2006 for decommissioning and abandonment of oil and gas infrastructure after caseation of operations in Nigeria. However, there is a need for stringent sanctions for infringement of the law. Therefore, there is a need for a specific law on decommissioning and abandonment of oil and gas assets or infrastructure in Nigeria with stringent penalties for non-compliance with the obligation. There are no regulations that particularly cover flares and vents in the case of gas flaring, although the Environmental Protection Law and its executive regulations generally apply to these activities. The executive regulations additionally specify the gas flaring operations’ permissible limits.

In response to concerns about climate change, the Qatari government has begun a number of initiatives to diversify its economy and reduce its near total reliance on the petroleum sector.

A polysilicon manufacturing plant in Qatar with a capacity of 8,000 metric tonnes per year, Qatar Solar Technologies, stated in March 2017 that it had begun producing polysilicon. When giving concessions, Qatar now seems to be abandoning the usual Exploration and Production Sharing Agreements and Development and Production Sharing Agreements. The tendency is for foreign petroleum corporations and Qatar Petroleum or its affiliates to participate in contractual joint ventures. ^37^

However, Nigeria has not taken significant or concrete steps to diversify from oil and gas to a viable production sector, particularly the auto-mobile fabrication plants, cement, fabric industry, the mining, agricultural activities, information and communication technology and other significance segments of the country’s economy to promote energy diversification by integrating renewable energy and other low carbon energy sources into the national energy mix to promote technology development and to encourage active engagement of the citizens particularly the youths in the development of low carbon energy technologies being the current global demand due to adverse contribution of fossil fuel utilisation to global climate change.

A polysilicon manufacturing plant in Qatar with a capacity of 8,000 metric tonnes per year, Qatar Solar Technologies, stated in March 2017 that it had begun producing polysilicon. When giving concessions, Qatar now seems to be abandoning the usual Exploration and Production Sharing Agreements and Development and Production Sharing Agreements. The tendency is for foreign petroleum corporations and Qatar Petroleum or its affiliates to participate in contractual joint ventures.

*Kuwait in perspective*

Kuwait is a major petroleum supplier and an OPEC member. Nearly half of Kuwait’s GDP, around 95% of exports and roughly 90% of government export revenue, are derived from petroleum. Kuwait now has a production capability of over 3.15 million barrels per day and has roughly 7% of the global oil reserves. The Kuwait Petroleum Corporation (KPC), a state-owned corporation, oversees the country’s petroleum industry. KPC has several corporations functioning under it which are known collectively as the “K companies”, for example, Kuwait Oil Company (KOC) is largely responsible for upstream operations. KPC manages the downstream petroleum operations. ^38^

KOC is the largest corporation in terms of revenue and is in charge of the production of petroleum. By 2040, the KPC plans to boost its capacity for oil production to 4.75 million barrels per day. Additionally, KPC declared plans to boost natural gas output to 4 billion cubic feet per day by 2030. Kuwait is one of the largest producers of oil and has one of the largest proven reserves of crude oil in the world, as previously stated. In the past, it flared off a considerable amount of its associated gases. It is also important to mention that KOC has made significant progress in recent years in reducing gas flaring in its operations. Kuwait has made efforts over the past decades to limit gas flaring in order to both cushion the effects of climate change, thereby executing a significant reduction in its carbon emissions, and provide its power plants with gas supplies to satisfy the country’s fast-rising energy demand. The residual gas flaring levels decreased by roughly 40% between 2014 and 2018. KOC contends that the reduction of associated gas flaring in Kuwait has been successful due to the following factors: a strong commitment from all levels of the corporation to make flare reduction a priority; significant financial investments in cutting-edge facilities and operations; joint efforts between KOC departments with downstream companies and customers to adapt to any unforeseen situations and limit the span of flaring; and a strong, beneficial collaboration with other organisations. ^39^ This is unlike Nigeria, a country that is blessed with a vast deposit of gas but still flares a large percentage of its associated gas during upstream petroleum operations despite the country’s challenge with attaining a stable power supply. Flared gas could be used to supply gas to turbines to generate electricity. However, flared gas has occasioned the loss of billions of dollars in revenue to the Federal Government of Nigeria. Gas flaring has been a concern due to its deleterious effects on the ecosystem, aquatic resources, and human health hence the need to combat the menace to promote a healthy and sustainable environment in Nigeria. ^40^

Kuwait also allotted a sizable budget to spend on technology initiatives that concentrate on reducing costs and improving operational processes since oil is the country’s main natural resource and there is a need to minimize the risk associated with declining oil prices. ^41^ The digitization of oil fields is a top priority for KOC. Utilizing the most cutting-edge technology to remotely monitor and operate oil fields at four separate sites in West Kuwait, North Kuwait, South and East Kuwait, Kuwait Oil Company completed pilot projects to digitalize oil fields. This positive initiative is the Kuwait Integrated Digital Field, and which comprises a thousand fields, and roughly half of KOC’s oil fields.

*Norway in perspective*

Norway is a leading example of a developed, efficiently run hydrocarbon economy. ^42^ The United Nations’ (UN) index of human development ranks Norway second after Switzerland as of 2022. The Norwegian Petroleum Act is consistent with the UN General Assembly Declaration on permanent sovereignty, and petroleum resources belong to the Norwegian people. In accordance with this clause, the Norwegian people will exercise complete control over their natural resources and use them to improve their welfare. ^43^ The people of Norway, as a unitary state, own the country yet the monarch is responsible for administering it under section of the PIA 2021 the Federal Government in Nigeria, on the other hand, owns all of the petroleum found within the territory, including the country’s continental shelf and exclusive economic zone. In Norway, Statoil is the commercial arm of the petroleum industry representing the state. ^44^ Statoil engages in exploration and production, natural gas supply, pipelines, decommissioning and research and development. In terms of transparency, Norway’s upstream petroleum industry has become more transparent as a result of Statoil, which operates like other for-profit oil corporations with no special status as a statutory conglomerate and where the government mainly serves a supervisory role and levies corporate taxes. The Norwegian Act also addresses domestic energy supply security through state participation. Norway dominates the retail product market because they prioritise its inhabitants before selling to other countries. The Norwegian energy policy supports the security of supply of petroleum directorate and aids the ministry with industry administration and regulatory issues. Policy concerns are separated from commercial management and regulatory challenges in this context. In contrast, the minister of petroleum in Nigeria fulfils commercial regulatory and policy functions.

## Discussion

Notwithstanding the huge potential offered by the PIA 2021, the study revealed the following gaps under the PIA 2021 which are not in line with the international best practices and which may impede the PIA’s effectiveness, among others:

▪ Gas flaring and Energy Transition: There is silence on the subject of energy transition in the PIA. Nigeria’s government plans to increase the production.
▪ Federal Control of the Petroleum Industry: NNPC Ltd. is established as a corporation under CAMA in accordance with Section 53 of the act in order to conduct commercial petroleum operations. Based on the phrasing of 53(3), it appears that the federal government will be the only shareholder in this company. State governments have no say in the administration or distribution of dividends from the corporation because the act stipulates that all shares are to be owned by the federal government and held by the ministry of finance on its behalf.
▪ The act also appears to undermine Section 162 of the constitution, which specifies that federal revenues are to be deposited into a Federation account and distributed to the different tiers of government per the constitution. In this situation, the NNPC Ltd. would benefit from being fully unbundled to increase its viability, but as it is, this has just bolstered NNPC Ltd.’s attitude of non-accountability.
▪ Host Community Development Trust: The PIA does not address the midstream operators’ contribution to the HCDT. However, the authority may issue regulations defining the contribution. The HCDT is to be financed at the rate of 3% of the operator’s upstream operations’ total operating expenses from the previous year. However, the PIA does not specify how much revenue midstream operators must put into the HCDT. The amount to be contributed is expected to be outlined in regulations issued by the Nigerian Midstream and Downstream Petroleum Regulatory Authority.
▪ The PIA’s impact on the existing fiscal framework is its most crucial component. The PIA reduces royalties and introduces a new HCT to replace the Petroleum Profits Tax. It is possible that the new royalty system, which will allow the government to profit from rising oil prices, may reduce the likelihood of future alterations to the fiscal system.
▪ One expected benefit of the PIA is a turnaround in Nigeria’s petroleum investment decline, attributable to the PIA’s potential to make the country more competitive relative to other petroleum-producing countries. The discovery of oil and gas in other regions of the world, notably West Africa, helps to explain the drop in Investment in Nigeria’s petroleum industry. Despite being one of the continent’s top producers and holding one of the highest reserves, Nigeria only received 4% of the $70 billion invested in Africa’s oil and gas sector between 2015 and 2019, as reported by KPMG. Data from Nigeria’s National Bureau of Statistics reveal that the industry represented 1.11 percent of total capital imports into the country in 2020, reflecting the decline in investment in the industry.
▪ The PIA also attempting to shift responsibility for securing facilities to the surrounding host communities, noted that Damage to oil and gas infrastructure due to vandalism or sabotage will be repaired using this host community entitlement. As an alternative to relying solely on upper government action, this system establishes measures to have communities police themselves.
▪ The PIA levies a one per cent charge on the wholesale price of petroleum products and threatens sanctions against petroleum corporations that misrepresent their financial standing. The new law, which replaces ten prior laws, prohibits residents from serving as trustees or executives for host communities. It stipulates that oil-producing regions must disperse revenues for capital projects within a fund established within 12 months.

### Key lessons on petroleum policies identified under Qatar, Kuwait and Norway regimes

Investors, especially those in the petroleum industry, in particular, will find that the policies of Qatar are not demanding. State ownership extends to all oil and gas reserves. There is a consensus that all-natural resources belong to the state. Domestic production accounts for the vast majority of Qatar’s hydrocarbon needs. Qatar Energy has the exclusive right to explore for, produce, invest in, and develop any hydrocarbons, including but not limited to petroleum and natural gas. Obtaining a licence or permit from Qatar Energy is a necessary step for any company interested in operating in the petroleum industry in Qatar. ^45^ Following a decision by Qatar Energy and the State of Qatar, leases, licences, and concessions for the exploitation of hydrocarbons are issued. The contractor agrees to provide Qatar Energy with a detailed overview of the planned petroleum activities before beginning such operations. Plans, location, production capacity, operating modes, engineering data, cost estimates, various computations, and other pertinent documents, information, and statistics are all included in this description. Rights in produced petroleum can be transferred to oil and gas companies through net production sharing methods under the Exploration and Production Sharing Agreement and the Development and Production Sharing Agreement. ^46^

Oil production in Kuwait is managed by the state-owned KPC. For KOC, digitising their oil fields is crucial to attaining their objectives. Pilot projects to digitalize oil fields were completed by KOC, which used state-of-the-art technology to remotely monitor and run oil fields at four different sites in West Kuwait, North Kuwait, South Kuwait, and East Kuwait. Kuwait has also set aside a substantial budget for technological efforts aimed at lowering costs and improving operational procedures, as oil is the country’s primary natural resource and there is a need to reduce the risk associated with falling oil prices. ^47^

When it comes to advanced, well-managed hydrocarbon economies, Norway is a prime example. The petroleum resources are the property of the Norwegian people, as stated in the Norwegian Petroleum Act and by the UN General Assembly Declaration on permanent sovereignty. As a result of taking full charge of their natural resources, the Norwegian people will be able to better their livelihood. Statoil is the state-backed commercial arm of Norway’s petroleum industry. Statoil is involved in a wide variety of industries, including exploration, production, natural gas supply, pipelines, decommissioning, and Research and development. In Norway, the gas pipelines are managed by a company called Gassco. While the Norwegian gas pipeline is a de facto monopoly, the country’s gas transport infrastructure is managed to increase productivity and decrease biases. Norway’s regime permits and encourages corporations that produce gas to sell it through a system that lets each licence sell its gas. This is not the situation in Nigeria, where the federal government maintains a monopoly. ^48^

Sections 4 to 7 of the Norwegian Petroleum Act provide for joint and multiple liabilities for polluters, ensuring that victims of environmental damage receive adequate compensation, and also sets rigorous obligations on licence holders. Statoil in Norway has grown more transparent by functioning like other for-profit petroleum corporations with no special status as a statutory conglomerate and where the government primarily functions in a supervisory role and charges corporate taxes. ^49^

The Norwegian Petroleum Act requires licensees or lessees to submit a two- to five-year decommissioning plan (including a thorough environmental impact assessment) when a licence expires, is relinquished, or the use of a facility is permanently discontinued. The Norwegian Act addresses domestic supply security through state engagement as well. Norway dominates the retail goods industry by prioritising sales to its citizens over sales to other countries.

It is obvious that, whereas all these nations have peculiar attributes that are in line with the global best practices in the petroleum industry, many of these provisions were not taken advantage of in the PIA 2021. Firstly, there is no state ownership of crude oil. This therefore possesses a peculiar disadvantage to investors in that the petroleum industry in Nigeria is regulated by several agencies. This is a major variance from what is obtainable in Qatar. Unlike Norway, Nigeria has not introduced the appropriate management of hydrocarbon economies. Likewise, Nigerian-owned corporations are not involved in the exploration, and production of crude oil, likewise, most of the natural gas supply are flared in a bid to increasing the exploration capacity of the crude oil. The Norwegian Act also contains adequate provisions for the compensation of victims of environmental degradation and transparency of the state-owned oil company. The Statoil company in Norway functions as a profit corporation which pays corporate taxes. The federal government merely performs a supervisory role in the administration of the company. The Norwegian Act also caters for supply security by prioritising the sale of retail goods by the industry to its citizens over sale to other countries. Kuwait on the final note, has policies invested at technological efforts which is aimed at lowering costs and improving operational procedures in the petroleum industry. ^50^

All these are the various advantages of the petroleum industry in Qatar, Kuwait and Norway, advantages which the PIA 2021 could have incorporated. However, these were not incorporated into the PIA, whether directly or otherwise. This further diminished the utility of the PIA, in the light of global best practices, using the three countries of Qatar, Norway and Kuwait examples for analysis. ^51^

Oil and gas administration by the government in Nigeria is also described as being purely utilitarian. The Nigerian government’s decision-making is heavily impacted by utilitarianism, as shown by an analysis of the country’s petroleum administration through the lens of utilitarian principles. Examining the effects of the Nigerian government’s utilitarian-influenced oil and gas management practises on the Niger Delta’s host communities, sheds insight into the problems that the region faces. Although progress has been slow in the Niger Delta region, the Federal Government controls most of the revenue on behalf of the country. Host Community members in the Niger Delta are directly affected by the petroleum industry’s lack of attention to sustainability, even when the majority of Nigerians gain from oil production. The administration of Nigeria’s oil and gas resources under the utilitarian approach did not effectively protect the human rights of the inhabitants of the Niger Delta. ^52^

This study, however, concludes that the sovereign or domanial ownership theory is the best applicable theoretical framework for producing positive applications of the PIA. This must however be reflected in such a way that the federal government does not assume absolute control and authority over natural resources (petroleum included) but must cut across to the state government, a reality of which is not currently practised in Nigeria.

## Conclusions

The PIA serves as a cornerstone upon which further progress can be made. While no legislation is perfect, Nigeria could benefit from commencing steps to work and improve various aspects of the PIA. The Niger Delta, which is responsible for most of Nigeria’s oil output, should not have to shoulder such a disproportionate share of the country’s oil revenues. This is inconsistent with the fact that 30% of NNPC Ltd.’s revenues, is set aside for frontier exploration. Despite the PIA’s many flaws, it did provide a strong foundation for the industry and is a step in the right direction. By highlighting some of the gaps in the PIA and petroleum operations in Nigeria, this study adds to the body of existing knowledge. The study will serve as a guide for the Nigerian government to reflect these practices by adopting consistent measures, and policies or making required modifications, while also identifying inadequacies of the existing regime and referencing best practices in other jurisdictions. The study will also aid lawmakers to make amendments to legislation, regulations, and policies when Nigeria’s Nationally Determined Contributions are not reflected in domestic laws. ^53^ Given the significance of including Nigeria’s international climate change obligations in domestic legislation like the PIA, regulators in Nigeria would be better prepared to enforce the current laws, rules, and policies and fulfil the country’s international obligations; therefore, in view of the findings above, and in relation to achieving the intendment of the PIA 2021, this study recommends as follows: The PIA should be amended to include provisions which cater for the underlisted:

a. petroleum corporations must be compelled to install suitable gas recapturing and/or re-injection facilities, similar to what is performed in developed countries. Gas flaring must also be outrightly prohibited with no exceptions. Since most infrastructure currently in place condone gas flaring, a timeline should be introduced by the amendment, within which all gas flaring infrastructure must be phased out.
b. To attain the desired objectives of the PIA, the HCDT resources must be appropriately administered. The PIA should therefore contain clear provisions on the appropriation of petroleum receipt quota for host communities, as well as the regulation of the actives of the HCDT. To address the different neglect, and environmental degradation of oil-bearing areas, the operator’s contribution of 3% of its yearly operating budget for the prior year in upstream petroleum activities to the HCDTF is incredibly low. At least ten per cent is feasible considering the irreversible environmental impact of operations. All parts of the country, but notably the host communities, should have their voices heard through a proper and thorough amending of the act. This should also include provisions to cater for collaborations between various operators in a host community, in order to prevent the duplication of efforts and cost maximisation in the development of host communities.
c. Also, the PIA mandates that 30% of the profits of petroleum receipts be invested in the exploration and development of frontier basins, or remote areas of land along the borders of some Northern states and Lake Chad where there is little confidence in the presence of crude oil. Even though it is not guaranteed, NNPC Ltd. is required to invest 30% of its profit in developing the frontier basins, which are new places where oil is anticipated to be found. It appears that this provision is unnecessary and ambiguous. Therefore, it has to be modified or expunged.
d. This study also addresses an important problem of why the host community should be held responsible for any act of sabotage on the corporations’ facilities located in the community, especially since most such acts of sabotage could be committed by strangers in connivance with some operational staff of the corporations. There is therefore the need to amend the PIA to include such provisions.
e. Likewise, the PIA needs to establish a system for resolving disputes between host communities and corporations. This will ensure that the interests of the operators as well as the host community is adequately catered for.

## Data Availability

No data are associated with this article.
