# Supplementary material for: An examination of the Petroleum Industry Act 2021: prospects, challenges, and the way forward
Source: F1000Res. 2023 Aug 16;12:551. Originally published 2023 May 25. [Version 2] doi: 10.12688/f1000research.132539.2 (PMC10468656; doi:10.12688/f1000research.132539.2)
Supplement: Peer review history

# Peer review: Reviewer response for version 2

**DOI:** 10.5256/f1000research.153263.r197203

**Published:** 2023-08-30

**Authors:** Azubuike Hope Amadi

**Affiliations:** 1 Universiti Teknologi PETRONAS, Bandar Seri Iskandar, Perak, Malaysia

I have gone through the updated work and it looks good to be indexed. Thank you once again for sharing your work, and I look forward to witnessing its impact.

Is the work clearly and accurately presented and does it cite the current literature?

Partly

If applicable, is the statistical analysis and its interpretation appropriate?

Not applicable

Are all the source data underlying the results available to ensure full reproducibility?

No source data required

Is the study design appropriate and is the work technically sound?

Yes

Are the conclusions drawn adequately supported by the results?

Yes

Are sufficient details of methods and analysis provided to allow replication by others?

Yes

Reviewer Expertise:

NA

I confirm that I have read this submission and believe that I have an appropriate level of expertise to confirm that it is of an acceptable scientific standard.

# Peer review: Reviewer response for version 2

**DOI:** 10.5256/f1000research.153263.r197204

**Published:** 2023-08-29

**Authors:** Enobong Mbang AKPAMBANG

**Affiliations:** 1 Ekiti State University, Ado Ekiti, Ekiti, Nigeria

Since the authors have acted on the corrections I pointed out, it should be approved now.

Is the work clearly and accurately presented and does it cite the current literature?

Yes

If applicable, is the statistical analysis and its interpretation appropriate?

Yes

Are all the source data underlying the results available to ensure full reproducibility?

Partly

Is the study design appropriate and is the work technically sound?

Yes

Are the conclusions drawn adequately supported by the results?

Yes

Are sufficient details of methods and analysis provided to allow replication by others?

Yes

Reviewer Expertise:

NA

I confirm that I have read this submission and believe that I have an appropriate level of expertise to confirm that it is of an acceptable scientific standard.

# Peer review: Reviewer response for version 1

**DOI:** 10.5256/f1000research.145469.r183238

**Published:** 2023-07-19

**Authors:** Enobong Mbang AKPAMBANG

**Affiliations:** 1 Ekiti State University, Ado Ekiti, Ekiti, Nigeria

The article discusses a contemporary issue relating to the  Nigerian petroleum industry. It examines the Petroleum Industry Act 2021 vis *-a-vis *the prospects, challenges, and the way forward in addressing the problems identified in the article. 

 The **background**, **methods**, and **results **are okay but there are some minor editorial corrections to be made on page 1 and other pages of the article. For instance under ' **Background**' line 8 should read: **Hence, the need for the PIA of 2021 to overhaul the industry to meet global standards; there are also some controversial provisions that pose challenges to its proper implementation.**

 Under **Results** on page 1, lines 5-6 should read .. **..the PIA was provided for weak institutions, which translates to weak implementation and enforcement of the law and thus, further widened the existing gap between law and reality.**

 The authors repeatedly used the word,  'act' to refer to the  PIA. It is suggested that they should use "Act" instead. 

**Literature Review**, paragraphs 1-4 (lines 1-20) on page five (5) of the article should be taken to and discussed as a continuation under the main Literature Review as the authors are still reviewing some available literature in the area.

 Under **ambiguous wording and imprecise language:** it is not correct for the authors to state that PIA failed to provide definitions of the terms: "frontier basin" and "host community". On the contrary, section 318 of the PIA, which is the interpretation section, defines the terms.  The said section defines "frontier basin" to "mean basins where hydrocarbon exploration activities has not been carried out or previous commercial discovery oil and gas have not been made or an area that is undeveloped and includes Anambra, Dahomey, Bida, Chad and Benue trough or as may be declared by the Commission through a regulation." On the other hand, the section goes further to define " host communities" as "communities situated in or appurtenant to the area of operation of a settlor, and any other community as a settlor may determine under chapter three" of the PIA. The authors are accordingly referred to the said section.

 Under **corruption** on page seven lines 1-2: the sentence is incomplete. The authors should also tell us what would happen if corruption is not tackled in the petroleum industry. 

 Under **Discussion **on pages 10-11, bullet three, lines 1-4: the authors should tell us how the PIA undermines section 162 of the 1999 Constitution of the Federal Republic of Nigeria (as amended).  It is not enough for the authors to merely assert that the PIA seems to undermine section 162 of the Constitution."

Is the work clearly and accurately presented and does it cite the current literature?

Yes

If applicable, is the statistical analysis and its interpretation appropriate?

Yes

Are all the source data underlying the results available to ensure full reproducibility?

Partly

Is the study design appropriate and is the work technically sound?

Yes

Are the conclusions drawn adequately supported by the results?

Yes

Are sufficient details of methods and analysis provided to allow replication by others?

Yes

Reviewer Expertise:

Oil and Gas Law; Constitutional Law; Comparative Constitutional Law; and Women and the Law

I confirm that I have read this submission and believe that I have an appropriate level of expertise to confirm that it is of an acceptable scientific standard, however I have significant reservations, as outlined above.

# Response

**Published:** 2023-07-19

**Authors:** OLUSOLA JOSHUA OLUJOBI

**Affiliations:** PUBLIC AND INTERNATIONAL LAW, Afe Babalola University, Ado Ekiti, Ekiti, Nigeria

**AUTHORS’ RESPONSES**

 We extend our deepest appreciation for the meticulous review and valuable suggestions provided by the esteemed reviewer. Your expertise and keen eye have undeniably enhanced the quality of our manuscript, and we are sincerely grateful for your time and effort.

 The article delves into a pressing contemporary issue, pertaining to the Nigerian petroleum industry, and meticulously examines the Petroleum Industry Act 2021 in relation to its prospects, challenges, and the way forward in addressing the problems identified in the article. We are deeply thankful for your insightful comments and suggestions that have undoubtedly strengthened the robustness of our work. May you and your family be blessed abundantly for your contributions. Once again, thank you, sir, for your unwavering support. We have diligently reviewed the manuscript, placing special emphasis on grammar and overall editing. Your feedback has been immensely valuable, and we truly appreciate it.

 1. Thank you for your discerning assessment of our manuscript. We are grateful for the positive feedback on the background, methods, and results. Moreover, we acknowledge the need for minor editorial corrections on page 1. As per your suggestion, we have revised the 'Background' section accordingly, and line 8 now reads as follows: "Hence, the need for the PIA of 2021 to overhaul the industry to meet global standards; there are also some controversial provisions that pose challenges to its proper implementation." Your attention to detail has been a tremendous asset, and we sincerely thank you for it.

 2. Your sincere commentaries and efforts to strengthen our manuscript are highly appreciated. We have duly corrected the mentioned section under the results on page 1, lines 5-6, as per your recommendation. It now reads: "The PIA was provided for weak institutions, which translates to weak implementation and enforcement of the law and thus, further widened the existing gap between law and reality." Thank you, sir, for your valuable input.

 3. We express our gratitude for your astute observation. We understand the significance of consistency in writing style. While we initially attempted to change the word "act" to "Act" as suggested, the Journal's in-house style ultimately prevailed. Nevertheless, we have duly rectified the word, 'act,' to 'Act,' referring to the legislation made by the National Assembly under the PIA. Your attention to detail and commitment to excellence are truly commendable, and we are thankful for your kind understanding.

 4. Your attention to the arrangement and continuity of the literature review is duly noted. We have incorporated your suggestion by taking paragraphs 1-4 (lines 1-20) on page five (5) of the article and seamlessly integrating them as a continuation under the main Literature Review section. Your keen eye for organization and clarity has been incredibly valuable, and we extend our heartfelt appreciation.

 5. We sincerely appreciate your diligent analysis of the ambiguous wording and imprecise language in our manuscript. Your observation regarding the definitions of "frontier basin" and "host community" in the PIA is entirely accurate. We apologize for the oversight and have duly corrected the statement. Section 318 of Nigeria’s PIA 2021, which serves as the interpretation section, indeed defines these terms explicitly. As you mentioned, "frontier basin" is defined as "basins where hydrocarbon exploration activities have not been carried out or previous commercial discovery oil and gas have not been made or an area that is undeveloped and includes Anambra, Dahomey, Bida, Chad, and Benue trough or as may be declared by the Commission through a regulation." Furthermore, "host communities" are defined as "communities situated in or appurtenant to the area of operation of a settlor, and any other community as a settlor may determine under chapter three" of the PIA. Your commitment to precision and accuracy is truly commendable, and we thank you for bringing this to our attention.

 6. Your thorough review has been invaluable in identifying an incomplete sentence. We have now completed the sentence in the section discussing corruption on page seven, lines 1-2. Moreover, we have elaborated on the consequences of corruption if left unaddressed in the petroleum industry. Corruption hampers transparency and deprives Nigerians of the benefits of their abundant resources, leading to a decline in their standard of living, social, energy security, and sustainability, thereby promoting mass poverty. Your commitment to clarity and comprehensive understanding is deeply appreciated.

 7. We are deeply grateful for your insightful suggestion regarding the discussion on pages 10-11. You have aptly pointed out that merely asserting the undermining of section 162 of the 1999 Constitution of the Federal Republic of Nigeria (as amended) by the PIA is insufficient. We have taken your advice and thoroughly explained how the PIA 2021 may undermine this constitutional provision. Specifically, by allowing the NNPCLTD to retain 30% from the production sharing contracts, profit sharing, and risk service contracts meant for frontier basins exploration, the PIA contravenes section 162 of the 1999 Constitution. This retention of 30% will reduce the oil revenue accruing to the Federal Government, states, and local governments, making it challenging for various government bodies to meet their statutory obligations. Furthermore, we have highlighted how other case study countries, which have embraced clean sources of energy and energy mixed to combat greenhouse gas emissions, have retained 0% for frontier basin exploration. These countries serve as valuable examples that could be emulated through constant reform under the PIA. Your exceptional insights and attention to legal aspects have significantly enriched our manuscript, and we sincerely thank you for it.

 8. In conclusion, we are immensely grateful for your dedication to excellence, your comprehensive review, and your valuable suggestions. Your contributions have undeniably strengthened our work, and we are indebted to you for your guidance and support. Thank you once again for your unwavering commitment to ensuring the quality and integrity of our manuscript.

# Peer review: Reviewer response for version 1

**DOI:** 10.5256/f1000research.145469.r183246

**Published:** 2023-07-14

**Authors:** Azubuike Hope Amadi

**Affiliations:** 1 Universiti Teknologi PETRONAS, Bandar Seri Iskandar, Perak, Malaysia

The research demonstrated a smooth flow, and in my opinion, the paper presents an intriguing and informative contribution within its scope, which will enrich the existing literature and enhance policy-making in Nigeria. However, across different sections, there were poor citations of facts in different paragraphs. If these are improved on, then the manuscript is good for indexing.

**Major points**: Citation of facts on paragraphs, Making a conclusion on % allocated for frontier basins without adequate comparison with the case studies.

**Minor points**: Typos, paraphrasing complex sentences, referencing the Acts introduced, 

 Below is the breakdown of my reviews on the different sections

**Introduction:** Citations needed P4 (Paragraph 4)

 Introduce the law first, before referring to it as "the law"

 At first instance, PIA should be referred to as Nigeria's PIA 2021

**Lit reviews:** Citations needed P4, P6

 P3: section 1 of the PIA confers... "to" the state.

 1 citation is not enough to justify a theory being introduced in this case.

**Results:** Citations needed P3, P10, P16, P20, P23

 P13: rephrase 1st sentence.

 Qatar and Norwegian Petroleum Acts were not referenced.

**Kuwait in perspective**: Citations needed P2

**Norway in perspective:** Rewrite 5th sentence

**Discussion:** cite 6th bullet point

**Conclusion:** Criticism of 30% to frontier basins would have been better compared with % kept for exploration by case studies used.

*Bullet d* **:** do you mean "should not be held"?

**Overall**

 The problem was identified, the method was clear, the findings were apt, and the suggested solutions were fair. However, evidence should be properly cited to avoid storytelling.

Is the work clearly and accurately presented and does it cite the current literature?

Partly

If applicable, is the statistical analysis and its interpretation appropriate?

Not applicable

Are all the source data underlying the results available to ensure full reproducibility?

No source data required

Is the study design appropriate and is the work technically sound?

Yes

Are the conclusions drawn adequately supported by the results?

Yes

Are sufficient details of methods and analysis provided to allow replication by others?

Yes

Reviewer Expertise:

Petroleum Engineering, Production, Petroleum Industry Policies & Politics

I confirm that I have read this submission and believe that I have an appropriate level of expertise to confirm that it is of an acceptable scientific standard, however I have significant reservations, as outlined above.

# Response

**Published:** 2023-07-14

**Authors:** OLUSOLA JOSHUA OLUJOBI

**Affiliations:** PUBLIC AND INTERNATIONAL LAW, Afe Babalola University, Ado Ekiti, Ekiti, Nigeria

- The research demonstrated a smooth flow, and in my opinion, the paper presents an intriguing and informative contribution within its scope, which will enrich the existing literature and enhance policy-making in Nigeria. However, there were poor citations of facts in different paragraphs across different sections. If these are improved upon, then the manuscript is good for indexing.

**AUTHORS' RESPONSE**

**Thank you very much for reviewing our paper, providing candid comments and suggestions to strengthen our work. We appreciate your efforts. The citations of facts in different paragraphs or across different sections have been improved upon, and we have implemented all the suggestions to make the paper better and suitable for indexing. We have thoroughly reviewed the manuscript, paying special attention to grammar and editing in general.**

  

- Major points: The citation of facts in paragraphs and making a conclusion on the allocation percentage for frontier basins without adequate comparison with the case studies.

**AUTHORS' RESPONSE**

**We have corrected the citation of facts in paragraphs and the conclusion on the 30% allocation for frontier basins without adequate comparison with the case studies. We have now included adequate comparisons with the case studies of other countries.**

**Frontier basins can be simply defined as areas where oil has not been found but there is a possibility of oil discovery, such as the Chad Basin, Sokoto Basin, Bida Basin, the Anambra platform, Calabar, and the Niger Delta areas, which have not been explored. Frontier basins hold significant reserves of oil and natural gas, with a projected volumetric ratio of 75% natural gas and 25% crude oil (Obaje, Adamu et al., 2022). However, the transition to low-carbon sources of energy has become crucial for achieving sustainable energy security. Therefore, it is essential for Nigeria to embrace clean energy sources, such as gas, and move away from fossil fuels (Olujobi et al., 2023).**

**According to Nigeria's Petroleum Industry Act (PIA), 30% of the revenue from production sharing contracts, profit sharing, and risk service contracts is allocated for frontier basins exploration. This allocation affects the revenue that should be remitted to the federation, and it will reduce the oil revenue and monthly allocation to various government entities, especially the state governments that rely on it for their day-to-day obligations. In comparison to the selected case study countries, which have embraced clean and mixed energy sources to combat greenhouse gas emissions and their adverse effects on climate change, the allocation of 30% of NNPC LTD's profit for exploration is relatively excessive. These case study countries have allocated 0% for frontier basin exploration.**

  

- Minor points: Typos, paraphrasing complex sentences, referencing the introduced Acts. Below is the breakdown of my reviews on the different sections. Introduction: Citations needed in P4 (Paragraph 4). Introduce the law first before referring to it as "the law." At first instance, the PIA should be referred to as Nigeria's PIA 2021. Literature reviews: Citations needed in P4, P6, and P3: section 1 of the PIA confers... "to" the state. One citation is not enough to justify the introduction of a theory in this case.

**AUTHORS' RESPONSE**

**We have meticulously corrected the typos, paraphrased complex sentences, and referenced the introduced Acts. The introduction has been completely reworked, introducing the law first before referring to it as "the law." The PIA is now referred to as Nigeria's PIA 2021.**

**We have provided the necessary citations in the Literature Review section, including in P4, P6, and P3 where section 1 of the PIA confers "to" the state. We have also included additional citations to support the introduction of theories in this case.**

**In the Results section, we have provided citations in P3, P10, P16, P20, and P23. We have rephrased the first sentence in P13 as suggested by the reviewer. We have also referenced the Qatar and Norwegian Petroleum Acts in the respective sections and provided citations under the Kuwait in perspective section (P2). Additionally, we have reworked the fifth sentence in the Norway in perspective section. Under the Discussion section, we have provided citations for the sixth bullet point.**

 The conclusion has been reworked as follows: The Petroleum Industry Act (PIA) 2021 allocates 30% of the revenue from production sharing contracts, profit sharing, and risk service contracts for frontier basins exploration in Nigeria. This allocation will reduce oil revenue and the monthly allocation to various government entities, especially state governments that rely on it for their day-to-day obligations. In comparison to the selected case study countries, which have embraced clean and mixed energy sources to combat greenhouse gas emissions and their adverse effects on climate change, the allocation of 30% of NNPC LTD's profit for exploration is relatively excessive. These case study countries allocate 0% for frontier basin exploration.

 The correction has been made for Bullet D, which now reads "should not be held."

 Thank you very much.

# Response

**Published:** 2023-07-18

**Authors:** OLUSOLA JOSHUA OLUJOBI

**Affiliations:** PUBLIC AND INTERNATIONAL LAW, Afe Babalola University, Ado Ekiti, Ekiti, Nigeria

We extend our sincerest gratitude for your dedicated effort in reviewing our paper. Your valuable feedback and candid comments have greatly contributed to enhancing the robustness of our work. We truly appreciate the time and expertise you have invested, and we wish abundant blessings upon you and your family. Once again, we express our heartfelt appreciation for your diligent contributions.

 We are delighted to inform you that we have diligently addressed all your suggestions, ensuring that the paper is now more suitable for publication and indexing. We have made significant improvements in the citation of facts, carefully placing them within paragraphs and across different sections. Additionally, we have rectified the issue of drawing conclusions without adequate comparison to the case studies by conducting thorough comparisons with the relevant countries.

 To provide clarity, we offer a concise definition of frontier basins as areas where oil has not yet been discovered, but there exists a possibility of finding oil. Notably, the Chad basin, Sokoto basin, Bida basin, the Anambra platform, Calabar, and the Niger Delta areas fall under this category, remaining unexplored. These frontier basins hold immense potential for vast reserves of oil and natural gas, with a projected volumetric ratio of 75% natural gas and 25% crude oil.

 In light of the urgent shift towards low-carbon energy sources for sustainable energy security, it is imperative for Nigeria to embrace cleaner energy sources, such as gas, and transition away from fossil fuels.

 Regarding the allocation of funds, the Nigeria Petroleum Industry Act (PIA) designates 30% of production sharing contracts, profit sharing, and risk service contracts to be allocated for frontier basins exploration. While this allocation supports the development of frontier basins, it is important to note that this revenue, which would otherwise be remitted to the federation, will reduce the overall oil revenue and monthly allocation to various government entities. State governments, in particular, heavily rely on these funds to fulfill their day-to-day statutory obligations. When compared to selected case study countries that have embraced clean and mixed energy sources, the allocation of 30% of NNPC LTD's profit for exploration in Nigeria appears relatively excessive. Notably, these case study countries allocate 0% for frontier basin exploration, emphasizing their commitment to combatting greenhouse gas emissions and addressing the adverse effects of climate change.

 We have meticulously addressed all typos, paraphrased complex sentences, and ensured proper referencing of the introduced Acts. Furthermore, the introduction has undergone a holistic rework, introducing Nigeria's PIA 2021 before referring to it as "the law."

 Additionally, we have provided the necessary citations in various sections of the paper, including the Literature Review (citations under P4, P6, P3), Results (citations under P3, P10, P16, P20, P23), and Discussion (citation for the 6th bullet point). The corrections you suggested, such as rephrasing the first sentence in P13, referencing the Qatar and Norwegian Petroleum Acts, and overhauling the fifth sentence under the Norway in perspective section, have all been meticulously implemented.

 Finally, we have reworked the conclusion, incorporating your suggestions. The 30% allocation from production sharing contracts, profit sharing, and risk service contracts, as stated in Nigeria's Petroleum Industry Act (PIA) 2021, will lead to a reduction in oil revenue and the monthly allocation to various government entities, particularly state governments. Moreover, in comparison to the selected case study countries that have embraced clean and mixed energy sources, the allocation of 30% of NNPC LTD's profit for exploration in Nigeria appears relatively excessive. This is due to the adverse effects of greenhouse gas emissions on climate change. In contrast, the case study countries allocate 0% for frontier basin exploration.

 We are pleased to inform you that all the corrections, including the necessary adjustments to the bullet point you highlighted, have been diligently implemented. It now reads, "should not be held." Once again, we extend our utmost gratitude for your invaluable feedback and guidance throughout this process.

 With warm regards
